# Antioxidant, Anticancer Activity and Phytochemical Analysis of Green Algae, *Chaetomorpha* Collected from the Arabian Gulf

**DOI:** 10.1038/s41598-019-55309-1

**Published:** 2019-12-11

**Authors:** Samina Hyder Haq, Ghaida Al-Ruwaished, Moudhi Abdullah Al-Mutlaq, Sundus Ali Naji, Maha Al-Mogren, Sarah Al-Rashed, Qura Tul Ain, Abir Abdullah Al-Amro, Adnan Al-Mussallam

**Affiliations:** 1 Biochemistry Department, College of Science, King Saud University, Riyadh, Saudi Arabia; 2 Microbiology and Botany Department, College of Science, King Saud University, Riyadh, Saudi Arabia; 3 The Wellman Centre for Photomedicine, Harvard Medical School, Boston, Massachusetts USA; 4 Chemist, Cosmetic Department, Saudi Food and Drug Authority, Riyadh, Saudi Arabia; 5 Department of Physics, The Islamia University of Bahawalpur, Bahawalpur, Pakistan

**Keywords:** Pharmaceutics, Cancer

## Abstract

Seaweeds are a group of marine multicellular algae; the presence of antioxidant phytochemical constituents in Seaweed *Chaetomorpha sp*. extracts has received attention for their role in the prevention of human diseases. This study explores the phytochemical constituents, antioxidant, and anticancer properties of the Cladophoraceae, *Chaetomorpha sp*. Energy dispersive x-ray spectroscopy (EDX), and Gas chromatography-mass spectrometry (GC/MS) were performed to study the chemical structure and chemical formula. Different concentrations of ethanol and aqueous extracts of *Chaetomorpha* were used to estimate antioxidant activity by 2,2-diphenyl-1-picrylhydrazyl (DPPH) radical scavenging activity and total flavonoid, phenolic, and tannins content assays. Anti-tumor activity against breast cancer cell lines (MCF-7 and MDA-MB-231) was assessed by 3-(4,5-Dimethylthiazol-2-cyl)-2,5-Diphenyltetrazolium Bromide (MTT) assay. The EDX analysis indicated the presence of oxygen, silicon, and calcium as dominant elements. Antioxidant assays indicated that the ethanol extracts of *Chaetomorpha* consisted of a total of 189.14 ± 0.99 mg QE/g flavonoid content, 21.92 ± 0.43 mg GAE/g phenolic content and 21.81 ± 0.04 mg GAE/g tannins content. The DPPH radical scavenging assay exhibited higher antioxidant activity IC_50_ (9.41 ± 0.54 mg/mL) in the ethanol extract. Moreover, it showed high anticancer activity by growth inhibition in the MDA-MB-231 breast cancer cell line and low IC_50_ (225.18 ± 0.61 µg/mL). GC/MS analysis revealed the presence of Dichloracetic acid (DCA) as the active antitumor constituent of *Chaetomorpha sp*.; other anticancer compounds identified were Oximes and L-α-Terpinol. The results revealed that the type of *Chaetomorpha sp*. studied here possesses very unique and novel constituents and active potent antitumor chemical constituents and it can act as a promising antioxidant and anticancer agent for future applications in pharmaceutical industries.

## Introduction

Marine algae (Seaweeds) are a group of marine multicellular algae, plentiful in minerals, vitamins, and polysaccharides. They are considered as a potential source of bioactive substances such as proteins, lipids, and polyphenols possessing potent antibacterial, anticancer, antioxidant, antifungal, and antiviral properties^1^.

Recently, the evaluation of antioxidant phytochemicals constituents in macro-algae extracts has received attention for their important role in the prevention of human diseases. The presence of antioxidant substances such as alkaloids, flavonoids, phenols, tannins, phlorotannin, terpenoids, pigments, glycosides, and steroids in algae was thought to act as a defense mechanism, protecting them against reactive oxygen species (ROS) resulting from harsh environmental conditions^2,3^. The presence of antioxidants in macro-algae protected the species’ structural components from environmental oxidative damage^4^.

ROS are produced endogenously from metabolic activity in the human body or exogenously from smoking, air pollutants, radiation, ozone, and industrial chemicals. ROS are stabilized by reactions which in turn cause cellular damage and the formation of carcinogenic DNA adducts. ROS is a major cause of human diseases involving the heart, brain, and various cancers. The consumption of antioxidants has shown to reduce the risks of getting these diseases^5^.

Breast cancer is the leading cause of death in women globally. Cancerous breast cells express survival factors that inhibit apoptotic cell death^6^. As described by Moussavou *et al*., (2014) “Employing natural or synthetic agents to prevent or suppress the progression of invasive cancers has recently been recognized as an approach with enormous potential”. Studies have shown that seaweed extracts could be powerful anticancer agents, apoptosis was detected in breast cancer cells that were treated by seaweed extracts thereby suggesting that seaweed could protect against breast cancer^6^. With the ever-increasing rate of Breast cancer incidence, there is a need to look for natural more effective cancer treatment that is not toxic to the normal cells. Developing a plant-based natural therapy for cancer treatment without harming the rest of the body is the greatest challenge in designing cancer drug therapy.

The green algae genus *Chaetomorpha* (Chlorophyte, Cladophorales) is characterized by unbranched heavy filaments. It includes about 70 species^7^, mostly rich in bioactive compounds, which makes them ideal for their use as dietary supplement and natural therapy for the treatment of diseases^8^. Some of these green macroalgae had exhibited cytotoxicity against number of cancer cell lines^9^. This study aimed to characterize and identify the active constituents of the green algae, *Chaetomorpha sp*. which was collected locally from the coast of the Arabian Gulf of Saudi Arabia. The study also explored the antioxidant and anticancer properties of this macroalgae.

## Results

### Characterization techniques

#### Scanning electron microscopy

**Figure 1:**
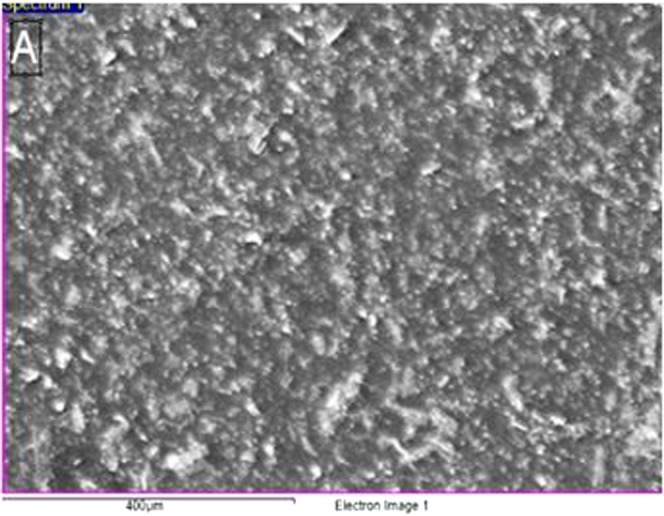
Scanning electron microscopy of *chaetomorpha sp*.

Field emission scanning electron microscopy (FESEM) was used to observe the microscopic morphology of *chaetomorpha sp*. Random-shape of *chaetomorpha sp*.was viewed under FESEM (Fig. 1).

#### Energy dispersive x-ray spectroscopy

**Figure 2:**
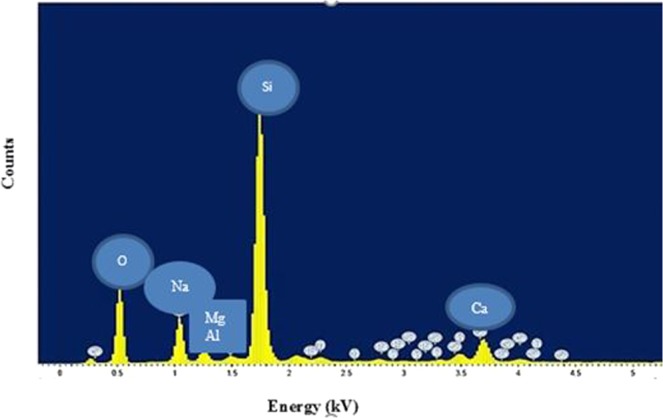
Energy dispersive x-ray spectroscopy of *Chaetomorpha sp*.

Energy dispersive x-ray spectroscopy (EDX) found the elemental composition of *chaetomorpha sp*. Figure 2 shows the EDX spectra. The highest oxygen percentage among all was observed (45%) and may be due to oxygen linkage. Silicon (Si) was found as a dominant element in the *chaetomorpha sp*. (32%). Sodium (Na) and calcium (Ca) (8.3%, 4.45%) respectively were observed as second and third ascendent elements. Moreover, a small percentage of magnesium (Mg), aluminum (Al), chromium (Cr), indium (In), stannum (Sn), and titanium (Ti) was found. Radioactive element radium (Ra)(1.2%) was also observed.

#### The total flavonoid, phenolic and tannin content

The total flavonoid, phenolic, and tannins content of aqueous and ethanol extract were estimated to evaluate phytochemicals content as shown in Table 1. The ethanol extract was registered as having a higher amount of flavonoid, phenolic, and tannins (189.14 ± 0.99 mg QE/g, 21.92 ± 0.43 mg GAE/g, and 21.81 ± 0.04 mg GAE/g, respectively) compared with the aqueous extract.

**Table 1:** Total phenolic, total flavonoids and tannins contents of ethanol and aqueous extracts of *Chaetomorpha sp*.

Solvent	Total flavonoid (mg QE/g)	Total Phenolic (mg GAE/g)	Tannins (mg GAE/g)
Ethanol	189.14 ± 0.99	21.92 ± 0.43	21.81 ± 0.04
Water	26.89 ± 0.77	2.15 ± 0.36	2.13 ± 0.006

Data were performed in triplicates and represented as mean ± SD.

#### 2, 2-Diphenyl-1-Picrylhydrazyl (DPPH)˙radical scavenging activity

DPPH˙Radical Scavenging assay was performed to assist in evaluating the antioxidant activity of *Chaetomorpha sp*. Lower IC_50_ indicates higher scavenging capacity. Ethanol extract of *Chaetomorpha sp*. showed higher antioxidant activity with lower IC_50_ (9.41 ± 0.54 mg/mL) compared to aqueous extract as shown in Table 2. The ethanol extract was significantly different compared with the aqueous extract with a p < 0.05. Ascorbic acid was used as the standard antioxidant which gave IC_50_ at 0.03 ± 0.01 mg/mL which was significantly different compared to the *Chaetomorpha* extracts, with a p < 0.05.

**Table 2:** Radical Scavenging Activity and Anticancer Activity of ethanol and aqueous extracts of *Chaetomorpha sp*.

Solvent	Antioxidant Activity IC_50_ (mg/mL)	Anticancer Activity IC_50_ (µg/mL)
Ethanol	9.41 ± 0.54	225.18 ± 0.61
Water	15.44 ± 0.98*	—
Ascorbic acid	0.03 ± 0.01	—

Data were performed in triplicates and represented as mean ± SD. Ascorbic acid as standard antioxidant.

*represent significantly different p < 0.05.

#### Anticancer assay

**Figure 3:**
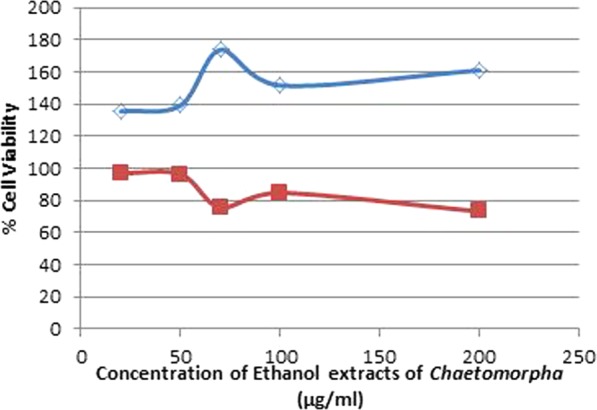
Effect of Different Concentration of Ethanol Extract of *Chaetomorpha sp*. on Breast Cancer Cell Lines. The blue line represents Cell viability percent of MCF-7 breast cancer cell line at a different concentration of *Chaetomorpha* ethanolic extract. Red line represents Cell viability percent of MDA-MB-231 breast cancer cell line at a different concentration of *Chaetomorpha* ethanolic extract.

MCF-7 and MDA-MB-231 breast cancer cell lines were used to study the cytotoxic effect of different concentrations (20–200 µg/mL) of ethanol and aqueous extracts of *Chaetomorpha sp*. on cell proliferation by 3-(4,5-Dimethylthiazol-2-cyl)-2,5-Diphenyltetrazolium Bromide (MTT) assay. Ethanol extract of *Chaetomorpha sp*. showed a significant effect on MDA-MB-231 but not on the MCF-7. It exhibited high anticancer activity by inhibition of cancer cell growth, with an IC_50_ value of 225.18 ± 0.61 μg/mL as shown in Table 2, which indicated the sensitivity of MDA-MB-231 breast cancer cell line against the ethanolic extract of Algae as shown in Fig. 3. The aqueous extract of *Chaetomorpha sp*. however, did not show any significant effect on MDA-MB-231 and MCF-7 breast cancer cell lines when compared to the control.

#### GC/MS analysis

**Figure 4:**
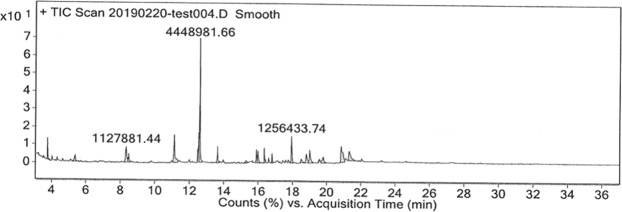
*GC-MS analysis of an ethanolic extract of Chaetomorpha sp*.

The ethanolic extract exhibiting significant antitumor activity was subjected to analysis by GC/MS technique. The major antitumor components found in that extract were Oxime, Lα-Terpinol and Dichloroacetic acid (DCA) as shown in Table 3 and Fig. 4.

**Table 3:** The major components found in the ethanolic extract of *Chaetomorpha sp*. based on GC-MS analysis.

	Name of the compound	RT min	Area%	Molecular Weight (g/mole)	Formula
1	*Oxime, methoxy-phenyl*	4.2	3.4599	151.165	C_8_H_9_NO_2_
2	1-Decene	5.3	0.7514	140.127	C_10_H_20_
3	1-Dodecanol	8.3	0.7134	186.33	C_12_H_26_O
4	*L-α-Terpineol*	8.4	21.1770	154.25	C_10_H_18_O
5	1-Hexadecanol	11.1	29.3378	242.447	C_16_H_34_O
6	2,4-Di-tert-butyl-phenol antioxadent33	12.4	100	206.32	[(CH_3_)_3_C]_2_C_6_H_3_OH
7	*Dichloroaceticacid,4hexadecyl ester*	13.6	15.3934	128.94	C_2_H_2_Cl_2_O_2_
8	Hexadecanoic acid	15.8	14.0434	256.4	C_16_H_32_O_2_
9	Neophytadiene	16.3	10.6915	278.524	C_20_H_38_
10	9-Octadecenamide	18.8	12.5969	281.477	C_18_H_35_NO

## Discussion

Marine algae produce a diverse array of compounds and chemicals that facilitate their survival and metabolism in extremely harsh and competitive environments. Research into the natural and unique bioactive compounds produced as a result of their biosynthesis of secondary metabolites has generated a renewed interest in the pharmaceutical industry. Their biodiversity and biodistribution made them unique in chemical composition and mineral content. Hence, they are a promising source of various therapeutic bioactive substances for the treatment of various diseases including cancer. In this study, we attempted to identify and characterize the major active chemicals and compounds found in *Chaetomorpha sp*. as well as to study its antioxidant and antitumor activity. A previous study^10^ found the major elements found in *Chaetomorpha* to be carbon and oxygen. Sulfur, potassium, sodium, and chlorine were found in minor amounts. In contrast, in this study, the major elements were found to be oxygen and silicon. The presence of silicon and oxygen as the major constituents of this algae make it unique from the point of view of its therapeutic and medicinal implications. Silicon bound to oxygen is soluble in water, can be easily absorbed and readily bioavailable to humans with biological activity^11^. However the exact biochemical and biophysical role of silicon is still unknown. As a result, there is a growing interest in the potential therapeutic effect of water-soluble silica on human health. Silicon has been shown to perform major roles in the structural integrity of nails, hair, skin, and total collagen synthesis and bone mineralization. There have been reports of direct involvement of silicon in reduced metal accumulation in Alzheimer’s disease, immune system health, and reduction in the risk of atherosclerosis^11,12^. The presence of radioactive elements, such as radium and titanium in *Chaetomorpha sp*.is noteworthy due to its therapeutic potential, especially in cancer treatment.

*Chaetomorpha sp*. is rich in polyphenolic compounds^13,14^. The presence of hydrophilic polyphenolic compounds such as phlorotannins, which are bipolar in nature could function as a major antioxidant, which helps the algae resist oxidative stress^15^. The presence of phenolic and flavanoids contributes to the antioxidants potential of *Chaetomorpha*^14^. The total free radical quenching DPPH assay revealed the antioxidant activity of IC_50_ 9.41 ± 0.54 mg/mL in ethanol extract and IC_50_ 15.44 ± 0.98 mg/mL for aqueous extract. These results were in agreement with previous studies that found higher antioxidants properties in ethanol solvents^15,16^.

There is a strong correlation between oxidative stress and the prevalence of cancer. Several *in-vivo* and *in-vitro* studies suggested that the administration of exogenous antioxidants may prevent free radical formation and damage to DNA and proteins thereby lowering the risk of developing cancer^17,18^. The prospects of using naturally occurring antioxidants alone or in combination with existing chemotherapy are an ideal strategy to combat tumor progression. Several studies regarding the cytotoxic efficiencies of various macroalgae and their potential anti-proliferative effect on the growth of cancer cells have been reported^18,19^. The antiproliferative and antitumor properties of *Chaetomorpha sp*. against two cancer cell lines MCF-7 and MDA-MB-231, observed in the results were of significance as it indicated its potential use as an antitumor therapy. Alpha-Terpineol was found to be a potential anticancer agent acting through suppressing NF-kb signaling in several breast cancer cell lines^20^. Steroidal oximes are gaining a lot of interest recently due to its antiproliferative and cytotoxic properties against cancer cell lines and considerable advances have been made in the development of an oxime functional group with the steroidal nucleus as an active anticancer molecule^21^. Most significantly the presence of DCA in the *Chaetomorpha sp*. has enormous potential to be used as anticancer drug therapy. Recent *in-vitro*/*in-vivo* rat study showed the efficacy of DCA in treating human lung, breast and brain cancer by inhibition of mitochondrial enzyme pyruvate dehydrogenase^22^. Recently oral DCA treatment for colon cancer has shown very promising results as a cytotoxic and cytostatic agent with an ability to maintain long term stability to advanced –stage cancer^23^. Our anticancer results supported the previous studies and further highlighted the importance of using algae as a therapeutic agent.

Further studies will be conducted against other types of cancer cell lines to understand the biochemical and molecular mechanisms of apoptosis of cancerous cells and the full therapeutic potential of the active ingredients present in this type of algae. As this is the first report of its bioactive constituents, the species characterization and the isolation of the bioactive compounds are currently in process.

## Conclusion

The use of natural and plant-based anticancer products is a useful tool to fight against the cancer cells due to their few or no side effects. Marine algae have already been used as a food supplement and antioxidants and currently, research on the health benefits of various types of Algae is gaining huge interest. This study demonstrated that the ethanol extract of *Chaetomorpha sp*. possessed higher Antioxidant and Anticancer activity compared to aqueous extract. Moreover, when the extracts were screened for Antitumor activity, MDA-MB-231 breast cancer cell lines were significantly affected by different concentrations of ethanol extracts of *Chaetomorpha sp*. This study demonstrated the anticancer activity of *chaetomorpha sp*. is due to the presence of several active potent Antitumor chemicals such as DCA, Oximes, and terpinol. Furthermore, it’s chemical composition consisting of silicon, calcium, and other precious metals make it an ideal therapeutic agent in novel drugs as well as nutritional supplements.

## Materials and Methods

### Chemicals

In the present study, sodium nitrite, sodium hydroxide, isopropanol, and Folin-Ciocalteau reagent were purchased from Winlab, U.K. Sodium carbonate, aluminum chloride, and polyvinylpolypyrrolidone were purchased from Loba Chemie, India. Gallic acid and 3-(4, 5-Dimethylthiazole-2-yo)-2, 5-diphenyltetrazolium bromide (MTT) were purchased from Sigma, USA. Quercetin was purchased from Sterilin England. DPPH was purchased from Atlantic and ascorbic acid from Avonchem, U.K. Dulbecco’s Modified Eagle’s Medium (DMEM), Trypsin-EDTA, Fetal Calf Serum (FCS), and antibiotic solution were purchased from UFC Biotech, KSA. MCF-7 and MDA-MB-231 breast cancer cell lines were donated by King Faisal Specialist Hospital and Research Center (KFSH&RC).

### Collection of algal material

The green alga, *Chaetomorpha* was collected in October 2017 at low tide time along the coast of the Arabian Gulf of Saudi Arabia. The algal material was washed under running tap water and allowed to dry in air. Finally, air-dried alga was powdered and stored at room temperature.

### Extract preparation

The dried powdered algae samples (*Chaetomorpha sp*.) were dissolved in either absolute ethanol or sterile autoclave water using a magnetic stirrer for 1 hour and then soaked at 25 °C and 4 °C respectively for 48 h with slow constant agitation. The mixture was sonicated 5 times for 30 seconds at (500 W, 25 kHz) using the Hielscher ultrasound sonicator and then filtered through Whatman No. 1 filter paper. The obtained filtrates were aliquoted and stored at −80 °C for further studies^24^. The stock solution of 10 mg/ml of *Chaetomorpha sp*. was used in all of the subsequent studies.

### Characterization techniques

To study the surface morphology of *Chaetomorpha* and elemental percentage, a scanning electron microscope (JEOL JSM-7610F FEG-SEM) was used.

### GC/MS analysis

The GC-MS analysis of an ethanolic extract of marine algae *Chaetomorpha* was carried out on Agilent technologies model 7890B GC coupled with a mass detector Agilent 5977 A GC/MSD. The Analytic column was Agilent J&W nonpolar column DB-5MS ((5%-Phenyl)-methylpolysiloxane, 30 m × 250 µm, 0.25 µm). Carrier gas helium (1 mL/min) was used to separate components. The different GC conditions were standardized as follows, injector parameters were injection volume (1 μL), while injector temperature was set at 280 °C (mass analyzer). During GC extraction the program of oven temperature was 1 min at 60 °C, increased to a temperature of 110 °C at a rate of 10 °C/min. Mass parameters were the following; the solvent delay time was 5 min. Transfer line temperature was 270 °C, Mass spectra were taken at an ionization mode with an electron impact at 70 eV; Ion source temperature was 230 °C, mass scan range was 50–400 m/z.

### Total flavonoid content

The total flavonoid content was estimated using the procedure described by Zhishen *et al*. 1999. A total of 1 mL of plant extracts were diluted with 200 µL of distilled water followed by the addition of 150 µL of sodium nitrite (5%) solution. This mixture was incubated for 5 minutes followed by the addition of 150 µL of aluminum chloride (10%) solution and then allowed to stand for 6 minutes. Next, 2 mL of sodium hydroxide (4%) solution was added and made up to 5 mL with distilled water. The mixture was shaken well and left for 15 minutes at room temperature. The absorbance of the reaction mixture was measured at 510 nm. The appearance of the pink color in the mixture showed the presence of flavonoids. The total flavonoids content was expressed as quercetin equivalent mg QE/g extract on a dry weight basis using the standard curve in the range of (0–200) mg/ml.^24^

### Total phenolic content

The total phenolic content was estimated using the Folin-Ciocalteau reagent. 500 µL of water and ethanol extracts were taken separately and it was made up to 1 mL of distilled water. Then 250 µL of diluted Folins- Ciocalteau reagent and 1.25 mL of 20% sodium carbonate (Na2CO3) was added. The mixture was shaken well and incubated in the absence of light for 20 minutes for a light pink color to develop. After incubation, the absorbance was measured at 735 nm. A calibration curve of gallic acid was constructed and linearity was obtained in the range of (0.25–10) mg/L. The total phenolic content in the plant extracts was expressed as mg of Gallic acid equivalent (mg GAE/g extract) by using the standard curve^24,25^.

### Estimation of tannins content

Tannin’s content was estimated by the method described by Siddhuraju & Manian 2007. A total of 500 µL of the extracts were taken in a test tube separately and treated with 100 mg of polyvinylpolypyrrolidone and 500 µL of distilled water. This solution was incubated at 4 °C for 4 hours. Then the sample was centrifuged at 5,000 rpm for 5 minutes and 20 µL of the supernatant was taken. This supernatant has only a simple phenolic free of tannins (the tannins would have been precipitated along with the polyvinylpolypyrrolidone). The phenolic content of the supernatant was measured at 725 nm and expressed as the content of free phenolic on a dry matter basis. From the above results, the tannins content of the extract was calculated as follows:

*Tannins* (*mg GAE/g extract*) = *Total phenolic* (*mg GAE/g extract*) − *Free phenolic* (*mgGAE/gextract*)^3^

### DPPH˙radical scavenging activity

The ability of algae extracts to scavenge the DPPH• radicals was assessed by using the method of Blois with some modifications^26^. About 0.2 mmol/L solution of DPPH• in ethanol was prepared, and 500 µL of this solution was added to different concentrations of the extracts (0.5–5 mg/mL). The mixture was shaken vigorously and allowed to stand for 30 minutes at room temperature. The control was prepared similarly but without the sample extracts and ethanol was used for the baseline correction. The changes in the absorbance of the algal samples were measured at 517 nm using the spectrophotometer. A lower absorbance value indicates a higher radical scavenging activity. Results were compared with different concentrations of standard antioxidant ascorbic acid (0.01–0.05 mg/mL). The ability of DPPH• radical scavenging activity was calculated by using the following formula:

$$DPPH\bullet scavenging\,effect( \% \,of\,inhibition)=(A0\,-\,A1)\ast 100/A0$$

where, A0 is the absorbance of the control, and A1 is the absorbance of the sample extracts. The IC_50_ (the milligram of extract to scavenge 50% of the radicals) value was calculated using linear regression analysis. The lower IC_50_value indicates greater antioxidant activity^3^.

### Anticancer assay

Breast cancer cell lines donated by King Faisal Specialist Hospital and Research Center (KFSH&RC) were used to test the activity of algal extract by MTT cell viability assay. The reduction of MTT was estimated by measuring the absorbance at 570 nm. The cells were cultured and maintained in DMEM supplemented with 2 mM L-glutamine, 10%FCS and 1% antibiotics (100 U/mL penicillin G and 100 mg/mL streptomycin). Both cell lines were plated separately in a flat-bottom 24-well plate (5 × 10^4^ cells/well) and treated with different concentrations of algal extract (0–200 µg/mL), in a humidified 5% CO_2_ atmosphere at 37 °C for 72 hours. After incubation 50 μL MTT solution (5 mg/mL MTT in PBS buffer)/well were added and the plate was shaken and incubated for 2 hours in a humidified 5% CO_2_ atmosphere at 37 °C. After incubation, 100 μL 0.04 N HCl with isopropanol were added and absorbance was measured by using microplate ELISA reader at 570 nm. The average of triplicate repeats was calculated for each concentration. The data were expressed as the percentage of relative viability:

$$Relative\,viability\,( \% )=Absorbance\,of\,treated\,cells/Absorbance\,of\,control\,cells\ast 100$$

Then, the value IC_50_ was calculated from the equation of the dose-response curve^5,27,28^.

### Statistical analysis

All data were expressed as mean values ± SD of triplicate. The mean values were analyzed by one-way ANOVA. Significant differences between the means of parameters were determined (p < 0.05).

## Data Availability

All data generated or analyzed during this study are available.
